# Comparing aperiodic activity in consumer-grade and research-grade EEG: Reliability and association with mathematical ability

**DOI:** 10.3758/s13428-025-02905-x

**Published:** 2026-01-06

**Authors:** Nienke E. R. van Bueren, Anne H. van Hoogmoed, Sanne H. G. van der Ven, Lisa M. Jonkman

**Affiliations:** 1 Behavioural Science Institute, Radboud Universiteit (Maria Montessorigebouw), Thomas van Aquinostraat 4, 6525 GD Nijmegen, The Netherlands; 2 Department of Cognitive Neuroscience, Faculty of Psychology and Neuroscience, Maastricht University, Maastricht, The Netherlands

**Keywords:** Aperiodic activity, EMOTIV EPOC X, BioSemi, Electroencephalogram (EEG), Cognitive development

## Abstract

Electroencephalography (EEG) provides valuable insights into brain development, but collecting high-quality data can be tedious, limiting its usability with children. This study evaluates the feasibility and reliability of EEG data acquisition in children with a wireless consumer-grade EEG headset (EMOTIV EPOC X), by comparing it to a research-grade system (BioSemi ActiveTwo), with a focus on aperiodic brain activity. The portability of the EMOTIV headset allows for EEG data collection in ecologically valid, real-world settings such as schools, enabling novel insights into brain activity during learning. We recorded EEG from 93 children (aged 9–10 years) using the EMOTIV headset, beginning with a 4-min resting-state measurement, followed by assessments of mathematical ability, visuospatial working memory, and verbal working memory, in a classroom environment. Aperiodic activity, thought to reflect fundamental aspects of neural excitability and cognitive processing, was extracted and its reliability compared across the two EEG systems. We further tested whether aperiodic activity recorded with EMOTIV predicts mathematical ability, replicating earlier research using research-grade EEG equipment. Our findings reveal that, similar to earlier findings, lower aperiodic activity was associated with higher math performance, supporting its role as a neural marker of cognitive ability. These results demonstrate the feasibility and reliability of using a consumer-grade mobile EEG headset to investigate individual differences in cognitive development in naturalistic contexts. This work opens up new opportunities for large-scale, school-based neurocognitive assessments and paves the way for personalized educational approaches based on neural profiles.

**Supplementary Information:**

The online version contains supplementary material available at 10.3758/s13428-025-02905-x.

## Introduction

Electroencephalography (EEG) is a widely used neuroimaging technique for studying cognitive development, providing valuable insights into neural mechanisms underlying various cognitive skills, such as numerical cognition, language processing, and visual perception (Beres, 2017; Hinault & Lemaire, 2016; Taylor & Thut, 2012). EEG measures brain activity with high temporal resolution, making it particularly useful for tracking cognitive functions in real time. However, research-grade EEG systems often require controlled laboratory conditions, lengthy setup procedures, and technical expertise, which limit their feasibility for large-scale or school-based studies (Grummett et al., 2015). Many setups still involve complex equipment, such as separate amplifiers and cables, which can be cumbersome and reduce the portability of the system. These logistical challenges, combined with the high cost of traditional EEG setups, make it difficult to study neural activity in real-world educational environments. In contrast, more affordable and user-friendly systems (e.g., headset-based EEG) offer increased mobility and faster setup, allowing for data collection from multiple participants simultaneously and in more naturalistic settings where learning occurs. Some of these systems now use water-based electrodes to reduce preparation time even further, minimizing the need for conductive gel and skin preparation. However, as these systems are still new, the reliability of these approaches still needs to be determined, in particular their use in newly developed measures in neuroscientific research. The aim of the present study is to validate the use of the portable EMOTIV EPOC X headset in measuring aperiodic brain activity in schoolchildren.

### Portable EEG in cognitive research

Portable EEG systems, such as the EMOTIV EPOC X headset, offer a promising alternative for studying brain activity in naturalistic environments, including schools (Dadebayev et al., 2022). Compared to traditional lab-based EEG, portable EEG improves ecological validity, reduces participant burden, and enables large-scale neuroimaging studies in educational settings. Moreover, children may find portable EEG headsets more comfortable and less intimidating, leading to higher compliance and better data quality. Some portable systems, including the EMOTIV EPOC X, use water-based electrodes to reduce setup time and avoid the need for conductive gel, further enhancing feasibility in applied contexts. However, water-based electrodes may be more susceptible to signal noise than traditional gel-based systems, potentially affecting data quality (Topor et al., 2021). Despite this limitation, the advantages of portability and usability make these systems particularly relevant for studying the neural mechanisms underlying cognitive functions such as mathematical ability, which plays a crucial role in academic achievement, career prospects, and daily functioning (Parsons & Bynner, 2005). Previous comparisons of consumer-grade saline-based EEG headsets with research-grade systems have focused primarily on event-related potentials (ERPs) and frequency-domain analyses. For example, Williams et al. (2020) validated the EMOTIV EPOC Flex for ERP and frequency analysis, and Badcock et al. (2015) tested a similar system for ERPs in children. These studies provide important evidence for the feasibility of portable EEG in cognitive neuroscience, but none have as yet examined the reliability of a consumer-grade headset in assessing aperiodic activity, a proposed marker of cortical excitation–inhibition balance (Donoghue et al., 2020; He, 2014). The present study therefore extends this line of work by focusing on aperiodic parameters in school-age children.

### EEG and mathematics

Strong mathematical skills rely on higher-order cognitive processes, including working memory, executive control, and decision-making (Dehaene, 2011), and are also associated with favorable life outcomes, such as academic achievement, increased employment opportunities, and greater financial stability (Butterworth et al., 2011; Parsons & Bynner, 2005). Because strong mathematical skills rely on higher-order cognitive processes, understanding their neural underpinnings may provide valuable insights into individual differences in learning and inform approaches to support individuals with difficulties in this domain. Recent EEG studies have increased our understanding of the neural basis of numerical cognition. Traditionally, these studies focused on periodic oscillatory activity, in particular theta (4–8 Hz) and alpha (8–13 Hz) oscillations, which have both been linked to arithmetic problem-solving and numerical reasoning (Artemenko et al., 2019). Additionally, ERPs, such as the P2p and N400 components, have been widely studied in relation to numerical processing and math performance (Ansari & Lyons, 2016; van den Berg et al., 2021). A growing body of research suggests that a different type of activity, aperiodic activity, is also linked to the neurophysiological basis of mathematical ability (Donoghue et al., 2020; Van Bueren et al., 2022, 2023; Voytek & Knight, 2015).

### Periodic versus aperiodic neural activity

Neural oscillatory activity can be classified broadly into periodic and aperiodic components. Periodic oscillations, which reflect synchronized neural firing such as theta synchronization and alpha desynchronization, are well documented in studies of mathematical processing (Soltanlou et al., 2018), with theta activity linked to working memory demands during numerical tasks (Grabner & De Smedt, 2012) and alpha activity reflecting arithmetic processing (Artemenko et al., 2019). In contrast, aperiodic activity, characterized by the 1/f slope of the power spectrum, has emerged as a novel neural marker of cognitive function (Voytek et al., 2015). Aperiodic activity is thought to reflect the balance between excitation and inhibition (E/I) in cortical networks (Donoghue et al., 2020; Gao et al., 2017; He, 2014). A lower spectral exponent (flatter slope) of aperiodic activity suggests greater cortical excitability, which has been associated with better cognitive performance, including mathematical ability (Chini et al., 2022; Waschke et al., 2021). Aperiodic activity may thus serve as a promising biomarker for tailored mathematical intervention strategies (Herzberg et al., 2024; Immink et al., 2021; Van Bueren et al., 2023). Furthermore, aperiodic activity varies across development and in neurodivergent populations, making it a candidate for capturing the dynamics of learning in general (Dakwar-Kawar et al., 2024; Van Bueren et al., 2022). However, most studies investigating aperiodic activity rely on relatively expensive, high-density electrode, lab-based EEG systems, which limit their feasibility for large-scale studies in real-world learning environments. While previous studies have demonstrated the feasibility of the EMOTIV headset for ERP measurements in both adults (De Lissa et al., 2015; Petit et al., 2023; Sabio et al., 2024) and children (Petit et al., 2020; Williams et al., 2021), its ability to reliably capture aperiodic activity remains unexplored. Given the growing evidence that aperiodic activity may serve as a neural marker of cognitive function, it is important to determine whether consumer-grade EEG headsets can provide measures of aperiodic activity that are comparable in reliability to those acquired with research-grade systems.

### Research objectives and hypotheses

In the present study, we aim to validate aperiodic activity recorded in children using a consumer-grade EEG headset (EMOTIV EPOC X). To do so, we compare the data with previously collected EEG data from a matched sample of children, similar in age and mathematical ability, measured with a research-grade EEG system (BioSemi; Van Bueren et al., 2022). We assess the reliability of aperiodic activity measurements from EMOTIV and BioSemi using equivalence testing to determine whether the systems yield comparable results. Demonstrating equivalence would provide evidence that the portable EEG headset captures aperiodic components with similar accuracy as the research-grade BioSemi. To assess reliability comprehensively, we employ multiple complementary approaches: (1) direct statistical comparison of aperiodic parameters between systems, (2) internal consistency analyses, and (3) a machine learning-based cross-validation analysis, designed to evaluate how well spectral features from each system can predict aperiodic estimates. In addition to spectral fitting and reliability analyses, we quantify several general data quality and stability metrics for each system (see Methods).

We also examine whether we can replicate the previously reported negative relationship between aperiodic activity and mathematical ability in children (Van Bueren et al., 2022) and explore whether working memory mediates this relationship. However, the absence of a significant association between aperiodic activity and working memory in the previous study makes it a less suitable means to validate a measure of aperiodic activity. Therefore, this mediation analysis is reported in the Supplementary Information. This secondary analysis provides further insight into the cognitive relevance of aperiodic activity, complementing our primary focus on the reliability of EMOTIV. Finally, we evaluate children's perceived comfort, ease of use, and fit while wearing the EMOTIV headset, as usability is a critical factor in the adoption of portable EEG systems for large-scale and ecologically valid studies in real-world educational settings (Xu & Zhong, 2018). Establishing the reliability of portable EEG headsets for measuring aperiodic activity and its relation to cognitive abilities helps make cognitive neuroscience more accessible, scalable, and impactful in childhood learning environments.

## Methods

### Participants

The EMOTIV sample consisted of 93 children (aged 9–10 years,* M* = 9 years, 7 months, *SD* = 0.64) attending grade 4, who were recruited from eight different Dutch primary schools through direct contact and information flyers. All parents gave active informed consent. There were no exclusion criteria for participation. Data from three children had to be removed (see “Resting-state EEG and preprocessing”). The final dataset, from 90 children, was compared to data from an existing dataset collected from 50 children using BioSemi (Van Bueren et al., 2022). The two samples did not differ significantly in age (EMOTIV: *M* = 9.69, *SD* = 1.16; BioSemi: *M* = 9.70, *SD* = 0.64; *U* = 1,880, *p* =.216) or in mathematical ability (EMOTIV: *M* = 101.97, *SD* = 27.57; BioSemi: *M* = 100.84, *SD* = 28.84; *t*(96) = −0.48, *p* =.626). The present study complies with the standards of the Declaration of Helsinki, and ethical approval was granted by the Ethical Advisory Committee of Radboud University (ECSW-2023-101, 12 October 2023). This study was preregistered on the Open Science Framework, and supplementary materials are available at https://osf.io/z52ux/?view_only=e76affb7cff14f0c93e3ca96ce60816d.

### Resting-state EEG

#### EMOTIV Setup

All EEG measurements using the EMOTIV EPOC X headset (channels: AF3, F7, F3, FC5, T7, P7, O1, O2, P8, T8, FC6, F4, F8, AF4) were conducted in a quiet room in the school. Each child's resting-state brain activity was recorded with a sampling rate of 128 Hz. During the recording, children were instructed to relax, keep their eyes open, and fixate on a cross displayed at the center of a laptop screen while sitting as still as possible to reduce motion artifacts. The setup was designed to minimize electrical interference, ensuring minimal disruption from sources such as electrical outlets and mobile phones. To align with the setup used in Van Bueren et al. (2022), the reference electrodes of the headset were repositioned from P3/P4 to the mastoids (M1/M2).

#### BioSemi setup

The previously collected dataset was acquired using a research-grade BioSemi ActiveTwo system with gel-based active electrodes (Van Bueren et al, 2022). EEG was recorded at a sampling rate of 512 Hz with online referencing to the common mode sense (CMS) and driven right leg (DRL) electrodes. Testing took place in a university laboratory in a sound-attenuated room, in contrast to the EMOTIV dataset, which was collected in schools. For the BioSemi ActiveTwo system, electrode offsets (DC potential differences relative to the CMS reference) were continuously monitored and kept below ±25 mV, following BioSemi guidelines. These offsets indicate stable electrode connections and prevent amplifier saturation in DC-coupled systems but do not represent electrode impedance measurements. Data were preprocessed and analyzed with the same pipeline as the EMOTIV recordings, ensuring comparability across systems. Since the BioSemi system provided more spatial coverage (32 electrodes), analyses for the present study focused on F3 and F4 to match the electrode locations available in the EMOTIV headset. For an overview of parameters and conditions under which the two datasets were acquired, see Supplementary Table S1.

#### Preprocessing

**Fig. 1:**
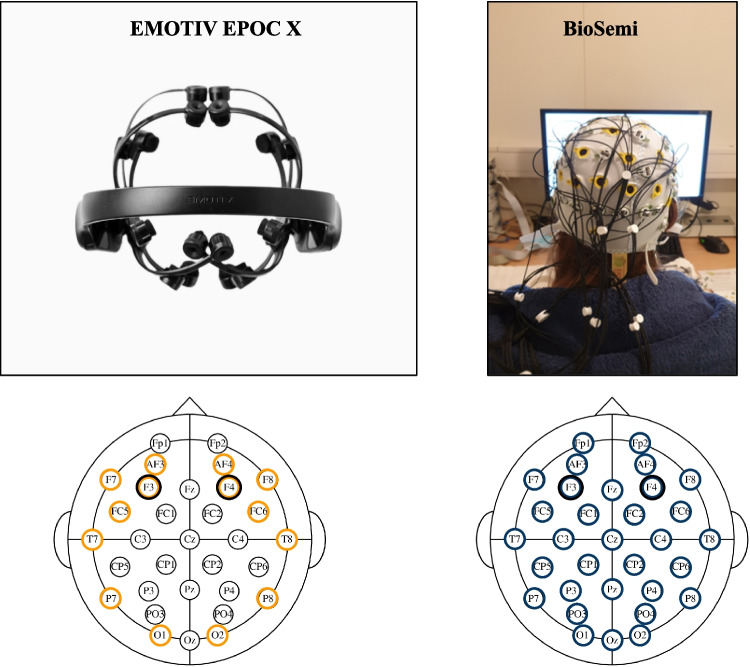
Comparison between a consumer-grade and a research-grade EEG system. The left panel shows the EMOTIV EPOC X headset, a consumer-grade EEG system with 14 electrodes (highlighted in orange). The right panel shows the BioSemi ActiveTwo system, a research-grade EEG setup with 32 electrodes (highlighted in blue). For the present analysis, only the frontal electrodes F3 and F4 (marked with a black border) were used

Figure 1 shows the difference in electrode setup between the EMOTIV EPOC X headset and the BioSemi system. For the present analyses of aperiodic activity, electrodes F3 and F4 were of main importance. EEG data preprocessing was conducted using the open-source toolbox EEGLAB (2024.0) running in MATLAB (R2023b; Delorme & Makeig, 2004) using the exact same preprocessing pipeline as Van Bueren et al. (2022). The preprocessing pipeline began with filtering, applying a 0.1 Hz high-pass filter to minimize slow drifts in the raw EEG signal and a notch filter at 50 Hz to eliminate line noise. Following filtering, all EEG data files were manually inspected for high-frequency artifacts caused by muscle movements, and excessively noisy data segments were removed. To further reduce artifacts, we performed independent component analysis (ICA) to identify and remove stereotyped artifacts, including eye movements, blinks, and heart rate activity. On average, 2.33 (*SD* = 0.68, min = 1, max = 4) components per participant were removed. Complete EEG recordings were excluded when more than 25% of the data had to be discarded, which was the case for three participants, leaving a final sample of 90 participants.

## Tempo test arithmetic

To assess mathematical ability, children completed a timed paper-and-pencil arithmetic test in a group setting (De Vos, 1992), identical to the test used in Van Bueren et al. (2022). The test consisted of five columns of arithmetic problems, each focusing on a different operation: addition, subtraction, multiplication, division, and a mixed-operation column. Problems were presented in order of increasing difficulty. Children had 1 min per column to solve as many problems as possible, resulting in a total test duration of 5 min. Research indicates that the TTR (TempoTest Rekenen; in English, Tempo Test Arithmetic) demonstrates good internal consistency, with Cronbach's alpha coefficients of 0.86 for the addition and subtraction subtests and 0.83 for the multiplication and division subtests (De Vos, 1992; Slot et al., 2016). Therefore, the total number of correct answers across all operations was summed to yield a total score, with higher scores indicating greater arithmetic fluency. The maximum number of problems children could solve was 200, resulting in a possible total score range of 0 to 200.

## Visuospatial and verbal working memory

Two computer-based tasks, the Lion game and the Monkey game, were administered in the classroom. These tasks were designed to assess visual-spatial and verbal working memory, respectively, in children (Van de Weijer-Bergsma et al., 2016). Each child completed the tasks individually on a school laptop. First, the Monkey game was administered. The Monkey game assesses verbal working memory by having children remember spoken words in backward order. After the words were presented orally, the participants were presented with a 3 × 3 matrix with nine written words, and they had to click on the words they had just heard in backward order. The task consisted of five levels. In the first level, children were presented with two-word trials; this increased with one extra word per level, ending with six words in the fifth level. Each level consisted of four trials. The mean proportion of correct answers over all trials was used as a measure of verbal working memory. Internal consistency has been shown to be satisfactory in the norm sample for the Monkey game (Cronbach’s *α* = .78 – .89 for different ages; Van de Weijer-Bergsma et al., 2016) and shows good concurrent and predictive validity.

The Lion game required children to find colored lions and remember their last location. In each trial, participants were presented with a 4 × 4 matrix of 16 bushes. Eight lions of different colors (red, blue, green, yellow, or purple) were presented consecutively at different locations in the matrix for 2,000 ms each. The same lion could appear in multiple locations. The task consisted of five levels, each comprising four trials. At level 1, children were told to remember the location of one specific colored lion where it had appeared last; at level 5, they had to remember five. After the initial display, the matrix reappeared with only the bushes visible. Children were then asked to indicate, by clicking, the last-seen location of a lion of a given color (e.g., “What was the last location of the red lion?”). The mean proportion of correct answers over all trials was used as a measure of visual-spatial working memory. This task has demonstrated satisfactory test–retest reliability (*ρ* = .71), high internal consistency (*α *= .86 and *α* = .90 for different ages), and good concurrent and predictive validity (Van de Weijer-Bergsma et al., 2016).

## Comfort questionnaire

After the individual EEG recording, children answered three multiple-choice questions to assess the comfort and usability of the EMOTIV headset. They were asked whether they found the headset comfortable to wear (yes or no), whether it was easy to put on (yes or no), and whether it felt too tight, too loose, or just right. These questions were not asked in the existing dataset collected with BioSemi, so a direct comparison across systems is not possible. Although our preregistration specified a comparison to an “average” comfort level, no validated normative data for such a comparison were available. Therefore, comfort ratings are reported descriptively.

## Procedure

**Fig. 2:**
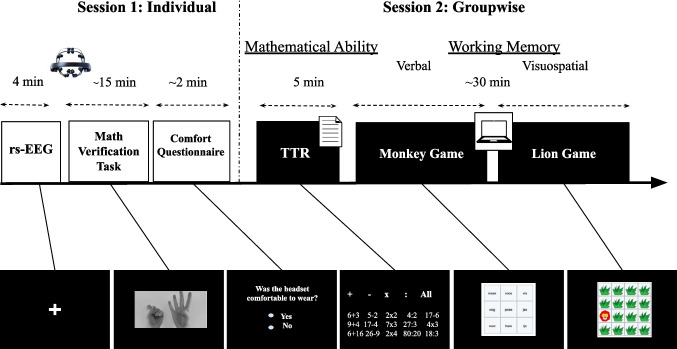
Overview of the experimental design. The study consisted of two separate sessions. In the individual session, children first completed a 4-min resting-state EEG (rs-EEG) recording using the EMOTIV EPOC X headset, followed by a ~15-min math verification task and a short comfort questionnaire about their experience with the EEG headset. In the group session, mathematical ability was assessed using the Tempo Test Arithmetic (TTR), and working memory was measured using two computerized tasks: the Monkey game (verbal working memory) and the Lion game (visuospatial working memory). The bottom row shows example stimuli for each task

Data collection took place at school in two separate sessions, one individual session (~19 min) and one group session (~35 min) (see Fig. 2). During the individual part, a 4-min resting-state EEG was recorded with the EMOTIV EPOC X headset, after which event-related EEG data were collected during a math verification task (outside the scope of the present study). A questionnaire assessing children’s ratings of the comfort of wearing the headset was administered at the end of the individual session. The group session was administered after all children had completed the individual session. During this session, all children performed the arithmetic fluency test (TTR), the Monkey game, and the Lion game together in the classroom.

## Aperiodic activity calculation

The fast Fourier transform (FFT) for the BioSemi (down-sampled to 128 Hz) and EMOTIV datasets was computed in MATLAB using Welch’s method, and the resulting power spectra were analyzed in Python (v3.8.10) using the FOOOF package (v1.0.0) over the 1–40 Hz frequency range. This approach decomposes the EEG power spectrum into its periodic and aperiodic components. Welch’s method was applied with the following parameters: window size = 1,024 samples (to improve frequency resolution), overlap = 75% (to maximize data retention while reducing variance). For aperiodic parameter extraction, EEG data from the left (F3) and right (F4) frontal electrodes were analyzed. Since frontal electrodes are known to be susceptible to muscle artifacts, we took precautions to minimize contamination: ICA-based artifact removal, visual inspection, and manual removal of segments containing artifacts to verify data quality. For spectral decomposition, the following FOOOFGroup settings were applied: peak_width_limits = [1,8], max_n_peaks = 5, no knee parameter was fitted to the data. Aperiodic activity was calculated as the average of the offset and the exponent. To assess model accuracy, the quality of the spectral fit was evaluated for each individual participant by computing the mean absolute error (MAE) and *R*-squared (*R*^2^) values.

## Data analyses

To evaluate the quality and reliability of EEG data collected using the research-grade BioSemi system and the EMOTIV EPOC X headset, several analyses were performed.

## Comparison of model fit quality across systems

We first assessed model fit quality for the aperiodic component of the power spectrum, as estimated by the FOOOF algorithm (fitting oscillations and 1/f; Donoghue et al., 2020), by computing the MAE and *R*^2^ values. These analyses were performed separately for F3 and F4, resulting in a total of two model fit evaluations per participant. Initially we preregistered the use of *t*-tests to compare spectral characteristics between the two systems (see Supplementary Information). However, the absence of significant differences is not a sufficient condition to establish equivalence (Lakens et al., 2018). Therefore, we additionally conducted equivalence tests using the two one-sided *t*-test (TOST) procedure. This approach explicitly tests whether the observed differences between two groups (in this case the EMOTIV and BioSemi EEG aperiodic scores) fall within a predefined equivalence margin, with a statistically significant test allowing us to conclude that the systems produce statistically equivalent measurements. Because there were significant deviations from normality in all measures (Shapiro–Wilk tests, *p* <.05), we conducted bootstrap-based equivalence testing (Davison & Hinkley, 1997), estimating the distribution of the mean differences between both datasets using 10,000 bootstrap samples and computing 90% percentile confidence intervals (CIs) for each metric. We defined the equivalence bounds (±Δ) a priori as ±0.0075 for MAE, ±0.0102 for *R*^2^ at F3, and ±0.0091 for *R*^2^ at F4. These bounds were based on the raw equivalence margins derived from prior TOST procedures on similar metrics. The assumption of equivalence was met if the entire 90% percentile CI fell within the corresponding predefined bound.

## Reliability of aperiodic activity measurements

To assess the reliability of aperiodic activity measurements within each system, we computed intraclass correlation coefficients (ICCs) to quantify the consistency of the extracted parameters (offset and exponent). For each participant, the resting-state EEG recording was divided into two equal-length segments, and FOOOF was applied separately to each half to obtain repeated estimates of the aperiodic parameters. Since the data from both systems were collected from separate groups of participants, a direct within-subject comparison between aperiodic components acquired with BioSemi and EMOTIV systems was not feasible. Therefore, we computed ICCs separately for each system and compared the resulting reliability metrics. ICCs were calculated using a two-way random-effects model for absolute agreement (ICC[2,1]; Koo et al., 2016). To test whether reliability differed between systems, we compared ICC values for EMOTIV and BioSemi using a nonparametric bootstrap procedure (10,000 resamples). For each system, electrode, and parameter (offset, exponent), participants were resampled with replacement and ICCs recomputed for each bootstrap sample. The distribution of the between-system difference in ICC (ΔICC = ICC_EMOTIV − ICC_BioSemi) was used to derive 95% confidence intervals.

## Cross-validation of the FOOOF model for EMOTIV and BioSemi

To evaluate the performance and generalizability of the FOOOF model for both datasets further, we implemented a cross-validation approach. The goal of this analysis was to assess how well the offset and the exponent extracted from each system could be predicted from each other and to determine whether one system yielded more consistent or learnable patterns than the other. This approach provides an indirect measure of data quality: more predictable (i.e., less noisy or more structured) data should result in better model performance. Prior to model selection, we performed an outlier analysis on the aperiodic parameters (offset and exponent) extracted from both datasets. Using the interquartile range (IQR) method, we identified and removed three extreme outliers in the BioSemi data at channels F3 and F4. These were removed to ensure a fair and robust comparison of model performance between systems. We then trained machine learning models to predict aperiodic parameters using spectral features. Each dataset (EMOTIV and BioSemi) was randomly split into a training set (80%) and a test set (20%), allowing us to assess how well the model generalizes to new, unseen data. The aperiodic values (offset and exponent) from electrodes F3 and F4 were first restructured into a long format, where each row represented a specific channel–component combination (e.g., F3 exponent) for a given system (EMOTIV or BioSemi). These two factors (channel–component and EEG system) were used as input features for the model. The target variable was the corresponding aperiodic value. A Gaussian process (GP) regression model was used to model the relationship between input and output variables (Rasmussen & Williams, 2006), and model accuracy was quantified using the mean squared error (MSE). In addition, to assess whether cross-validation errors in the EMOTIV data were disproportionately driven by a few extreme cases, we performed a sensitivity analysis. For each electrode and parameter, we excluded the three largest absolute errors (and any severe outliers defined by Tukey’s rule, Q3 + 3 × IQR) and recomputed mean absolute error (MAE). The change in MAE was quantified with a nonparametric bootstrap (10,000 resamples) to derive 95% confidence intervals (see Supplementary Table S5).

## General data quality and stability

In addition to spectral fitting and reliability analyses, we quantified general data quality metrics for each system: average power in the 49–51 Hz band (line noise) and 0.1–1 Hz band (low-frequency drift), percentage of retained segments (mean and range across overlapping electrodes), and participant exclusion rates. General data quality metrics were computed on the preprocessed, filtered data used for reliability analyses. Figure S3 shows spectra after applying a 50 Hz notch filter for visualization only. As filtering mainly attenuates line noise without altering relative between-system differences, this approach provides an appropriate estimate of overall signal quality. Summaries are provided in Supplementary Table S2; Power spectral density (PSD) overlays up to 80 Hz are shown in Supplementary Fig. S3. To assess whether signal quality changed over time, we compared noise metrics between the first and second halves of each resting-state recording. Using the split-half PSDs, we computed mean power in the 49–51 Hz band (line noise) and the 0.1–1 Hz band (low-frequency drift) for electrodes F3 and F4, averaged within each half. Paired Wilcoxon signed-rank tests were used to evaluate within-session changes. For BioSemi, electrode offsets were additionally monitored online and remained within ±25 mV throughout the recordings.

## Aperiodic activity and mathematical ability

To test whether the relationship between aperiodic activity and mathematical ability reported in Van Bueren et al. (2022) could be replicated using data collected with the EMOTIV system, we conducted bivariate Pearson correlation analyses in R. Aperiodic activity was operationalized as the average of the offset and exponent parameters extracted from frontal electrodes F3 and F4. This composite was then correlated with children’s mathematical ability scores to evaluate whether lower aperiodic activity was again associated with higher math performance, as previously observed with a research-grade EEG system. As preregistered, all continuous predictors (mathematical ability, visuospatial and verbal working memory scores, and aperiodic EEG parameters) were *z*-standardized prior to analysis to ensure comparability across measures. In addition, we conducted a simple mediation analysis (Hayes model 4) with working memory added as a mediator, using PROCESS in R (version 4.0.1), for which the results are mentioned in the Supplementary Information.

## Perceived level of comfort of the EMOTIV EPOC X headset

To evaluate the perceived comfort of the EMOTIV EPOC X headset, we visually inspected the distribution of participants’ responses on the comfort rating scale.

## Deviations from the preregistration

Our preregistration included exploratory analyses of oscillatory frequency bands (theta, alpha, beta). Although preregistered as exploratory, these analyses were not directly relevant to our main research question and would have extended the scope of the manuscript considerably. To maintain clarity and feasibility within the revision process, we therefore did not pursue them further. The preregistration also included a *t*-test approach and a mediation analysis, which were subsequently amended: Equivalence testing replaced *t*-tests, because our main interest was not in testing differences, but rather similarities between the systems. The mediation was moved to the Supplementary Materials (see preregistration amendment). Our preregistration also specified a comparison of headset comfort ratings to an “average” level. Since no validated normative data for such a comparison were available, we reported the comfort ratings descriptively instead. These deviations are documented for transparency and are in line with recent recommendations on preregistration reporting (Lakens, 2024; Schubert et al., 2025).

## Results

### Comparison of EMOTIV EPOC X and BioSemi aperiodic activity

**Fig. 3:**
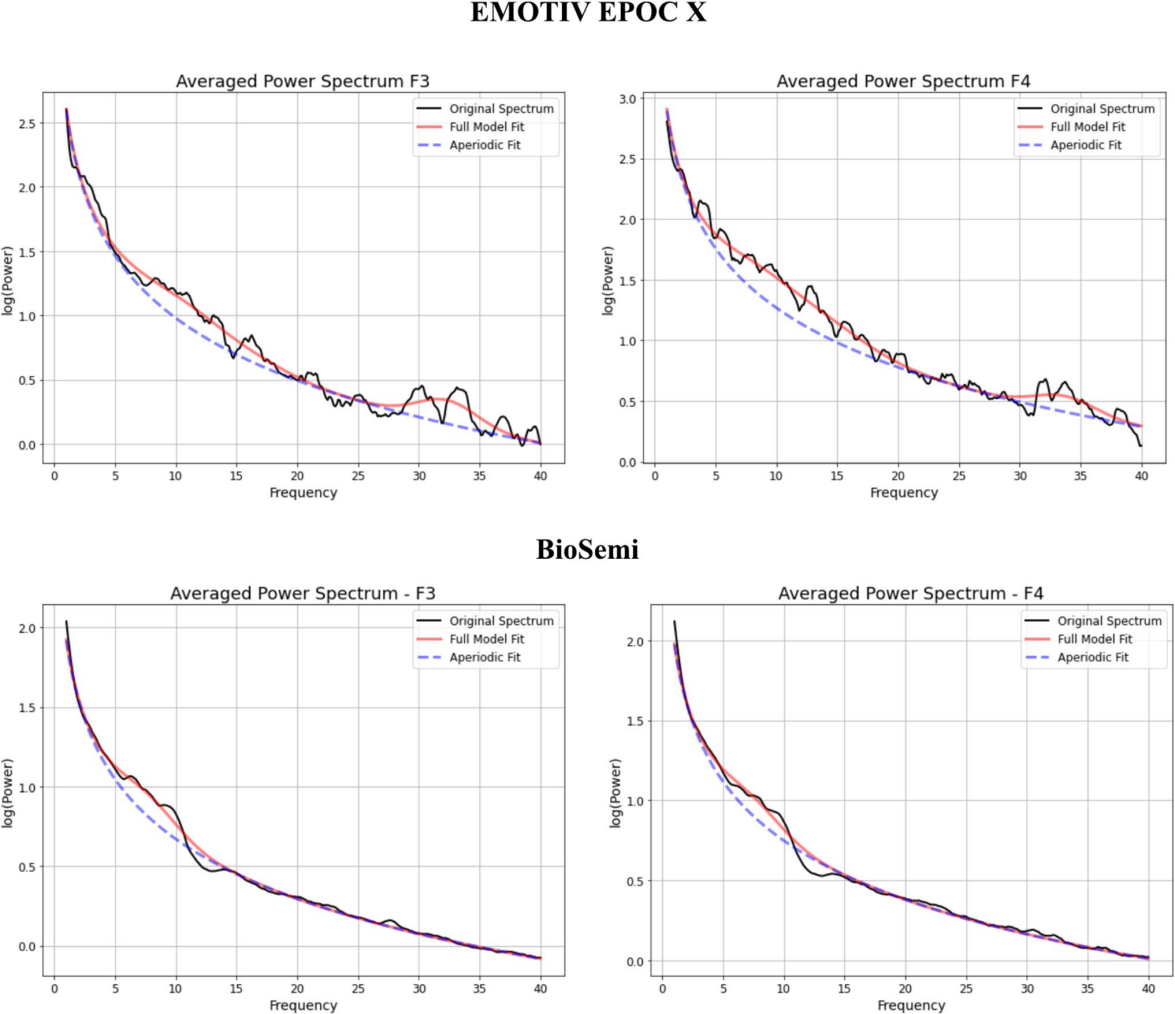
Comparison of power spectra between the EMOTIV EPOC X (top row) and BioSemi (bottom row) EEG systems. Comparisons are made for electrodes F3 (left) and F4 (right) with spectra averaged across *n* = 90 children in the EMOTIV dataset and *n* = 50 for the BioSemi dataset. The black line represents the original power spectrum, the red line indicates the full model fit, and the blue dashed line shows the aperiodic fit.

We compared the aperiodic activity data recorded with the EMOTIV EPOC X and BioSemi EEG systems by computing power spectra at frontal electrodes F3 and F4. Next, we modeled the aperiodic components, specifically the exponent and model fit indices (*R*^2^ and MAE), using the FOOOF algorithm. This modeling was performed separately for each electrode and EEG system. The power spectra for both systems exhibit similar shapes, with a characteristic 1/f slope, reflecting the aperiodic component of neural activity. The full model fit (red line in Fig. 3) follows the original spectrum closely in both datasets, and the aperiodic fit (blue dashed line in Fig. 3) captures the exponential decay of power across frequencies. Notably, while the general spectral structure is preserved across the datasets of both systems, visual inspection revealed minor differences in lower-frequency components and a small peak around 30–35 Hz in the EMOTIV data that was not present in the BioSemi spectra. This could be due to higher sensitivity of the EMOTIV headset to hardware-related noise in the higher-frequency range. Closer inspection indicated that the ~30 Hz bump was systematic across the EMOTIV sample, rather than driven by a small subset of participants. To investigate this further, we recomputed PSDs up to 80 Hz (see Supplementary Fig. S3). This analysis revealed that the bump is part of a harmonic noise component, most likely reflecting environmental or hardware interference (e.g., monitor refresh rate or wireless transmission) rather than neural oscillations. Because the FOOOF model explicitly separates periodic peaks from the aperiodic slope, this artifact did not substantially bias the estimation of aperiodic parameters.

## Comparison of model fit quality across systems

The *R*^2^ values indicated strong model fits, with values ranging from 0.760 to 0.998 (see Supplementary Information). Due to significant deviations from normality in our measurements, we employed a bootstrap equivalence test (with 10,000 resamples) to assess whether the mean *R*^2^ and MAE values between the EMOTIV and BioSemi systems were statistically equivalent. Table 1 presents the results of these equivalence tests for each metric (*R*^2^ and MAE at F3 and F4), including the mean value for each system, the difference between systems, the 90% CI, and the predefined equivalence bounds for the four metrics (see Methods section for justification). In all cases, the 90% CI exceeded the equivalence bounds, meaning we could not conclude that the systems are statistically equivalent. Interestingly, the EMOTIV system consistently showed slightly higher *R*^2^ and MAE values compared to BioSemi, indicating marginally better model fit but also slightly greater absolute error. However, none of these differences were statistically significant at the conventional alpha level of .05, as all CIs included values close to zero. Thus, while the systems cannot be deemed formally equivalent, the observed differences were small and may not be meaningful.

**Table 1:** Outcomes of the bootstrap equivalence test

**Metric**	**EMOTIV (*M*)**	**BioSemi (*M*)**	**Mean difference**	**90% CI**	**Equivalence bound**	**Equivalence outcome**
*R*^2^ F3	0.987	0.969	0.009	[−0.370e^−3^, 0.020]	±0.010	Not equivalent
*R*^2^ F4	0.981	0.966	0.015	[0.006, 0.025]	±0.009	Not equivalent
MAE F3	0.073	0.058	0.014	[0.008, 0.021]	±0.007	Not equivalent
MAE F4	0.071	0.060	0.011	[0.004, 0.017]	±0.007	Not equivalent

*Note.* Mean difference refers to the average difference in performance between systems. The 90% confidence intervals (CI) were calculated using bootstrapping. Equivalence bound indicates the predefined smallest effect size of interest. Equivalence outcome reflects whether the entire CI fell within the equivalence bounds, indicating statistical equivalence

## Reliability of aperiodic activity measurements

To evaluate the reliability of aperiodic activity metrics within each EEG system, we computed the intraclass correlation coefficient (ICC) for the offset and exponent parameters extracted from the power spectra at electrodes F3 and F4. For each participant, the resting-state EEG recording was divided into two equal-length segments of approximately 2 min, and FOOOF was applied separately to each half. This provided repeated measures of each aperiodic parameter per individual, allowing for a valid estimation of within-system reliability. The results showed excellent reliability for both the BioSemi and EMOTIV systems, particularly for the exponent parameter. For BioSemi, ICCs ranged from 0.902 to 0.919 across channels and parameters (F3 offset: ICC = 0.919, 95% CI [0.861–0.953]; F3 exponent: ICC = 0.919, 95% CI [0.862–0.953]; F4 offset: ICC = 0.902, 95% CI [0.834–0.943]; F4 exponent: ICC = 0.913, 95% CI [0.852–0.949]). The EMOTIV system yielded similarly strong reliability for the exponent parameter (F3 exponent: ICC = 0.938, 95% [0.907–0.959]; F4 exponent: ICC = 0.911, 95% CI [0.862–0.943]) and somewhat lower reliability for the offset parameter (F3 offset: ICC = 0.807, 95% CI [0.721–0.869]; F4 offset: ICC = 0.760,95% CI [0.657–0.835]). To ensure comparability across datasets, we repeated all EMOTIV reliability analyses in a random subsample of 50 participants (see Supplementary Information, Tables S3 and S4). Results from this subsample closely matched those from the full *N* = 90 sample, confirming that the larger sample size did not artificially inflate reliability estimates. These findings indicate that both EEG systems provide consistent aperiodic estimates across segments, with exponent values being particularly stable.

Finally, to test whether offset reliability differed between systems, we bootstrapped the difference in ICCs (10,000 resamples). Bootstrap comparisons of ICCs between systems (Supplementary Table S5) indicated no significant differences for exponent parameters at either electrode (all *p* ≥.46). For the offset parameter, EMOTIV showed lower ICCs than BioSemi, particularly at F4, but the bootstrap confidence intervals for ΔICC included zero (F3: ΔICC = −0.11, 95% CI [−0.22, 0.00], *p* =.050; F4: ΔICC = −0.14, 95% CI [−0.35, 0.02], *p* =.111), suggesting that these differences were not statistically reliable.

## Cross-validation of the FOOOF model for EMOTIV and BioSemi

To evaluate the quality and reliability of the aperiodic parameter estimates derived from both EEG systems further, as part of our broader aim to assess the reliability of EMOTIV compared to BioSemi, we conducted a model-based cross-validation analysis. Given the relatively constrained distribution of the BioSemi data, a random forest (RF) model yielded the best performance for this dataset (MSE = 0.003 after outlier removal). For the EMOTIV data, which showed greater variability and a more nonlinear structure, a Gaussian process regression (GPR) model performed best (MSE = 855.89). While this MSE appears much larger, the median absolute error (MAE) for EMOTIV was 0.45, indicating that the large MSE was primarily driven by a few extreme deviations rather than poor overall model performance. Note that MSE and MAE are not directly comparable in magnitude: the large EMOTIV MSE was inflated by a few extreme outliers, whereas the median absolute error (MAE = 0.45) reflects the typical error for most cases. These results suggest that although EMOTIV data are more variable, they can still be modeled reliably with appropriate algorithms. In sum, this analysis shows that both EEG systems can yield usable aperiodic estimates if model choice and data preprocessing are tailored to the properties of each system. While BioSemi produced more consistent and narrowband data (requiring outlier correction), EMOTIV data contained greater variability but could be modeled well using nonlinear methods that account for this uncertainty.

EMOTIV exhibited higher error variability than BioSemi across electrodes and parameters (EMOTIV MAE = 0.20; BioSemi MAE = 0.10). Excluding a small number of extreme EMOTIV cases (1–3 per combination) slightly reduced MAE (ΔMAE = 0.02–0.03), but the 95% bootstrap confidence intervals all included zero (e.g., F3 offset ΔMAE = 0.024, 95% CI [−0.025, 0.088], *p* =.41). BioSemi showed similar minor, nonsignificant reductions (ΔMAE = 0.01–0.014, all *p* >.14). These findings indicate that EMOTIV’s broader error distribution is not driven solely by a few outliers but reflects a generally higher variability in the recordings (see Supplementary Table S6).

## General data quality and stability

In addition to spectral fits, we quantified general data quality indices for each system. EMOTIV recordings showed higher power in line-noise (49–51 Hz) and low-frequency (0.1–1 Hz) bands, as well as a greater proportion of rejected segments compared to BioSemi, although overall signal quality remained sufficient for reliable estimation of aperiodic parameters (for a summary of these data see Supplementary Table S2). Although Table S2 lists smaller numeric values for EMOTIV, these reflect higher 49–51 Hz power on the log scale, consistent with the visual spectra shown in Supplementary Fig. S3. We also examined within-session stability by comparing noise indices in the first and second halves of the recordings (Supplementary Table S7). These analyses showed no significant changes over time for either system, aside from a small nonsignificant increase in low-frequency drift in EMOTIV. Small but statistically significant decreases in both low-frequency and line-noise power were observed across halves for EMOTIV, and a similar decrease in line-noise power was observed for BioSemi (see Supplementary Table S7). These changes were minor in magnitude, indicating overall stable signal quality over time. Participant exclusion rates were low and similar across systems (3/93 for EMOTIV; 2/52 for BioSemi). PSD overlays up to 80 Hz further illustrate the relative noise profiles of the two systems (Supplementary Fig. S3). Differences in general data quality metrics between the systems showed the same pattern when we repeated the analysis with a random subsample of *N* = 50 children from the EMOTIV dataset to match the sample size of the BioSemi data, supporting the robustness of these findings.

## Aperiodic activity and mathematical ability

A secondary aim was to explore whether we could replicate the finding of a negative relation between children's aperiodic activity and their mathematical ability as reported in our earlier study in which EEG was assessed with a research-grade system (Van Bueren et al., 2022). Here we first explored the bivariate correlations between aperiodic activity (assessed as the average of the offset and exponent parameters at electrodes F3 and F4), mathematical ability, and working memory capacity. Aperiodic activity was significantly and negatively correlated with mathematical ability (*r* = −.23, *p* =.034, 95% CI [−.41, −.02]), replicating the direction and strength of effects found in Van Bueren et al. (2022) who used a moderation model for a sample of children of the same age and with similar mathematical ability as the children in the current study (*β* = −0.36, *p* =.009). To compare the strength of the correlations across datasets formally, we conducted a Fisher’s *Z*-test, which indicated no significant difference between the EMOTIV and BioSemi samples (*Z* = −1.11, *p* =.27; see Supplementary Information). In both datasets, aperiodic activity was not significantly correlated with either verbal working memory (EMOTIV: *r* = −.05, *p* =.661, 95% CI [−.25, .16]) or visuospatial working memory (EMOTIV: *r* = −.12, *p* =.251, 95% CI [−.32, .09]). By contrast, both verbal working memory (EMOTIV: *r* =.32, *p* =.002, 95% CI [.12, .50]) and visuospatial working memory (EMOTIV: *r* =.30, *p* =.005, 95% CI [.09,.48]) were positively associated with mathematical ability, a pattern that was also observed in the BioSemi dataset (see Supplementary Information). Given the absence of a significant association between aperiodic activity and working memory, we did not include the mediation analysis in the main text but report it in the Supplementary Information. Taken together, these results indicate that the negative association between aperiodic activity and mathematical ability is observed in both datasets, albeit more clearly in EMOTIV, while the relationship with working memory is absent. This suggests that the aperiodic–math link is independent of working memory capacity.

## Perceived level of comfort of the EMOTIV EPOC X headset

To assess the perceived comfort of wearing the EMOTIV EPOC X headset as indicated by the children themselves, we evaluated children’s responses to a short questionnaire. Results indicate that 65.60% of the children responded “yes” to the question “Was the headset comfortable to wear?” with yes/no answer options (Supplementary Table S8). Additionally, 48.90% of children responded “yes” to the question “Was the headset easy to put on?” (Supplementary Table S9). Regarding the fit of the headset, most children (78.90%) rated it as “just right,” while a smaller proportion (16.70%) indicated that it was “too tight,” and 4.40% found it “too loose” (Supplementary Table S10). These findings suggest that while the headset was tolerated by all children (with no dropouts during setup or recording), comfort ratings were moderate, and ease-of-use ratings were evenly split. Although the percentages are moderate, and children had no point of comparison with other EEG systems, it is notable that all participants completed the full EEG session without dropout, supporting its feasibility for use in developmental EEG research.

## Discussion

In the present study we investigated the reliability of aperiodic activity measurement in a consumer-grade EEG headset (EMOTIV EPOC X) and compared the results to data collected earlier with a research-grade system (BioSemi). Our results indicate that the EMOTIV EPOC X headset provides comparable reliability to BioSemi in measuring aperiodic parameters (offset and exponent). Furthermore, low aperiodic activity was weakly associated with high mathematical ability, replicating findings from a previous study (Van Bueren et al., 2022). Finally, questionnaire responses indicated that a small majority of children found the EMOTIV headset comfortable, whereas ease-of-use ratings were mixed. Nevertheless, all participants were able to complete the recording session without dropout, supporting the feasibility of using consumer-grade EEG in school-based research.

## Reliability of aperiodic activity in consumer-grade versus research-grade EEG

Our visual inspection of the power spectra from the EMOTIV and BioSemi systems revealed generally similar spectral profiles, indicating that both systems capture the characteristic 1/f aperiodic activity. However, there were minor differences. Notably, the EMOTIV data exhibited some high-frequency noise, most evident as a small peak between 30 and 35 Hz. This feature appears to be a systematic artifact rather than neural activity and likely reflects the greater susceptibility of the EMOTIV headset to hardware- and environment-related noise. Consumer-grade, water-based headsets lack the active shielding and stable gel-based contact of research-grade systems, making them more sensitive to mains interference, monitor refresh harmonics, and wireless transmission artifacts (Ratti et al., 2017). By contrast, the BioSemi ActiveTwo system is wired and uses gel-based active electrodes with actively shielded leads, which substantially reduce such sources of noise. Although these spectral differences did not compromise the overall model fit (both systems showed strong *R*^2^ values and low mean absolute errors), they may contribute to additional variability in the extracted aperiodic parameters, which in turn could reduce the sensitivity of the measurements to detect individual differences or meaningful associations with cognitive outcomes. Visual inspection of the spectra also revealed that oscillatory activity in the 5–15 Hz range showed higher power in the BioSemi dataset compared to the EMOTIV dataset (Fig. 3). This band includes alpha and lower-beta activity, which are known to contribute to cognitive processing. The clearer oscillatory peak in the BioSemi data likely reflects their higher signal-to-noise ratio, allowing periodic components to be more readily distinguished. In contrast, the noisier EMOTIV recordings may obscure such features, consistent with prior reports that water-based electrodes yield lower SNR than gel-based electrodes (Topor et al., 2021). Although the present study focused on aperiodic activity, these qualitative differences in periodic components highlight an important avenue for future work comparing the capacity of consumer-grade systems to capture oscillatory activity.

Equivalence testing showed that the two systems were not statistically equivalent on model fit quality metrics (MAE and *R*^2^), indicating that even though both performed reasonably well, EMOTIV and BioSemi do not yield interchangeable predictive performance for these parameters. It is important to emphasize that a failure to confirm equivalence does not automatically prove that the systems produce meaningfully different measurements. Instead, it indicates that the current data, affected by inherent variability and measurement noise, did not provide sufficient estimates to conclusively rule out differences. In other words, while our model fits are robust, the uncertainty around the differences in spectral parameters is too large to claim statistical equivalence.

Intraclass correlation coefficients (ICCs) indicated strong to excellent within-session reliability for both systems. While BioSemi showed higher reliability for the offset parameter, EMOTIV demonstrated higher reliability for the exponent, particularly at electrode F3. This pattern suggests that under the current recording and preprocessing conditions, both systems are capable of producing stable aperiodic estimates, but that reliability may vary depending on the specific parameter of interest. The BioSemi system exhibited greater variability in ICC values, which could reflect increased sensitivity to individual differences or susceptibility to artifacts. Notably, these differences may stem from the systems' underlying electrode technologies rather than impedance variability. While BioSemi uses gel-based active electrodes applied until impedance is below a predefined threshold, EMOTIV EPOC X uses water-based electrodes, which are faster to apply but potentially more prone to signal degradation (Topor et al., 2021). Prior studies have reported that water-based systems can exhibit reduced SNRs and increased susceptibility to high-frequency noise compared to gel-based systems (Topor et al., 2021). These differences in electrode type and signal stability likely contributed to the spectral noise observed (the small peak around 30–35 Hz) in the EMOTIV data and should be considered when interpreting the reliability and precision of parameter estimates. Nonetheless, the more consistent ICC values observed for EMOTIV, particularly for the exponent, suggest that this system may be less affected by individual variability or artifacts than BioSemi under the current recording conditions.

Additionally, to test whether this pattern was driven by a few extreme cases, we performed an outlier sensitivity analysis. Excluding the largest EMOTIV errors slightly reduced MAE (ΔMAE = 0.02–0.03), but the bootstrap confidence intervals included zero (Table S5), confirming that the greater variability reflects general noise in the EMOTIV recordings rather than being attributable to a small number of outliers.

## Aperiodic activity and mathematical ability

In line with Van Bueren et al. (2022), our results indicated that low aperiodic activity (i.e., a flatter exponent and lower offset) was significantly associated with better mathematical ability in children. This negative relationship supports the hypothesis that increased cortical excitability, reflected by a flatter 1/f slope, may enhance numerical processing efficiency (Voytek et al., 2015; Waschke et al., 2021). Notably, our finding that lower aperiodic activity is associated with better mathematical ability aligns with previous work (Van Bueren et al., 2022) and with other studies that reported similar negative associations between aperiodic parameters (such as a flatter exponent or lower offset) and cognitive performance, including math ability (e.g., Kalamala et al., 2024; Waschke et al., 2021; Voytek et al., 2015). While the correlation in our study was statistically significant, the effect size was somewhat smaller compared to our previous study with BioSemi (Van Bueren et al., 2022). However, a Fisher’s *Z*-test indicated that the difference in correlation strength between the two datasets was not statistically significant, suggesting that the association is robust across systems despite variability in effect size estimates. One possible explanation is the difference in electrode location: the earlier study by Van Bueren et al. (2022) focused exclusively on electrode Fz, whereas the present study used a composite measure from F3 and F4, as Fz is not available in the EMOTIV headset. However, other factors may also have contributed, such as differences in sample size, noise, or variation in signal quality between systems. Importantly, while the two samples (BioSemi and EMOTIV) were independent, they were matched in age and mathematical ability, both assessed with the Tempo Test Arithmetic (TTR), which strengthens the validity of the comparison. Moreover, the current study included a larger sample (*N* = 90) than our previous work (*N* = 50), which typically increases statistical power and the stability of observed relations. Despite the higher noise observed in the EMOTIV spectral data, particularly at higher frequencies, the replication of the association between aperiodic activity and math ability is encouraging. This suggests that the aperiodic broadband components, such as the 1/f slope and offset, are relatively robust to noise, especially compared to narrowband oscillatory measures or event-related potentials (ERPs). These findings support the potential of using water-based, off-the-shelf headsets such as the EMOTIV EPOC X for scalable, real-world measurements of aperiodic neural activity. Given recent evidence linking aperiodic activity to learning and cognitive skills (Kalamala et al., 2024; Waschke et al., 2021; Van Bueren et al., 2022; Voytek et al., 2015), this opens the door to applying portable EEG in ecologically valid settings such as classrooms.

## Comfort and usability of consumer-grade EEG in children

One critical factor in the widespread adoption of portable EEG for educational research is participant comfort. In our study, all children completed the EEG session, and no participants dropped out during headset placement, suggesting good overall feasibility. Approximately two thirds of children (65.60%) reported that they found the EMOTIV headset comfortable, while children's ratings of ease of use were evenly split (48.90% yes vs. 51.10% no). In addition, most children (78.90%) rated the fit as “just right.” These mixed findings suggest that although the EMOTIV headset was generally tolerated, it cannot be unequivocally described as comfortable or easy to use. Without a direct comparison with comfort ratings of other caps used with research-grade systems, it remains unclear whether the EMOTIV headset is preferable to traditional gel-based systems/caps regarding comfort. Nonetheless, the absence of dropouts and the reasonable levels of tolerance support its potential feasibility for school-based research, provided that further design improvements (e.g., adjustable straps, softer padding) are considered. Nonetheless, anecdotal reports suggest that children often find lab-based EEG setups more intimidating due to extensive preparation time and gel application, which may favor the use of consumer-grade EEG in real-world educational settings.

## Limitations

One limitation of our study is that EMOTIV and BioSemi data were collected from different participant samples, rather than from the same individuals using both systems. While our results suggest comparable group-level reliability, future studies should aim to collect simultaneous dual-system recordings within the same group of children to directly assess within-subject reliability. Another potential limitation concerns the signal quality of the EMOTIV recordings. Although our spectral fits were generally robust, the EMOTIV data showed slightly more high-frequency noise. This may not be an inherent limitation of the system itself but could have been the result of practical factors during setup. For instance, unlike elastic EEG caps that are available in multiple sizes and can be individually adjusted, the EMOTIV headset has a fixed frame, which may not fit all children's head shapes equally well. This could lead to suboptimal electrode contact and greater susceptibility to environmental noise. Additionally, although EMOTIV provides visual indicators of signal quality during setup, no quantitative impedance values were available, and electrode–skin contact may have varied across participants. Incorporating more detailed measures of signal quality (e.g., impedance or noise floor estimates) in future work would help clarify to what extent setup procedures or headset design contribute to signal variability. In sum, while the quantitative goodness-of-fit measures are promising, the visual and qualitative differences in the higher-frequency range suggest that caution is warranted when directly assessing within-subject comparability of the two EEG systems. Additionally, the EMOTIV EPOC X is a self-contained headset with predefined electrode locations, which imposes constraints on spatial coverage and may limit its applicability in studies requiring signals from specific or high-density scalp regions. It is important to note, however, that EMOTIV also offers more flexible systems, such as the EMOTIV Flex, equipped with (water- or gel-based) electrode caps that do allow for custom electrode placement. Whereas these caps require more set up time, it may offer advantages in studies that require higher spatial precision or are particularly sensitive to signal noise. Although the EMOTIV Flex system is more expensive than EPOC X, it is usually still cheaper than research-grade battery-powered systems with portable amplifiers. In short, researchers should carefully consider these hardware-specific trade-offs when designing studies that require flexibility in electrode placement or dense spatial sampling.

### Data quality considerations for water-based, consumergrade EEG

Multiple indicators converged to show that EMOTIV recordings are more susceptible to environmental and low-frequency noise than BioSemi recordings. Likely contributors include water-based electrode contact (which can dry or shift during recording), as also suggested by our session-stability analyses showing significant decreases in both low-frequency and line-noise power across halves (see Supplementary Table S3). Other contributing factors are absence of active shielding of leads, and greater exposure to ambient electronics in school settings (e.g., monitor refresh harmonics, mains-related interference, or radio-frequency-related artifacts). Consistent with this, EMOTIV showed a small systematic bump near ~30 Hz in the average spectrum and slightly lower offset ICCs (though not significantly different from BioSemi in bootstrap tests), alongside higher low-frequency power and more segment rejections. These differences did not undermine the reliability of aperiodic parameters, but they can obscure periodic peaks and modestly reduce sensitivity for individual-difference associations. Researchers planning to use consumer-grade, water-based headsets should (i) pre-register quality control thresholds for line noise and drift, (ii) monitor stability across halves and re-wet sensors as needed, (iii) minimize nearby electronics (battery-powered laptops, avoid or dim high-refresh monitors, distance from Wi-Fi routers), (iv) keep recordings brief to limit drying, (v) oversample modestly to offset expected segment loss, and (vi) consider gel-based/actively shielded systems when oscillatory measures are the primary endpoint.

## Implications

Our study demonstrates that consumer-grade EEG (EMOTIV EPOC X) can reliably measure aperiodic activity, with comparable spectral estimates to research-grade EEG (BioSemi). Furthermore, we provide additional evidence that aperiodic activity is associated with mathematical ability, supporting its potential role as a neural marker for cognitive performance. These findings have significant practical implications. Establishing the reliability of portable EEG for measuring aperiodic neural dynamics paves the way for large-scale neurocognitive research in educational settings. By allowing for cost-effective, scalable, and ecologically valid measurements, consumer-grade EEG systems can help bridge the gap between laboratory neuroscience and real-world learning environments. In contrast to prior validation studies focusing on ERPs and oscillatory measures (Williams et al., 2020; Badcock et al., 2015), our work provides an independent replication using a different system and outcome measure. By demonstrating reliable measurement of aperiodic activity in 9–10-year-old children in their school environment, this study broadens the scope of portable EEG validation and highlights the potential of consumer-grade headsets for large-scale, ecologically valid research on neural markers of learning. However, we also note that comfort and usability ratings for the EMOTIV headset were mixed, with only a small majority of children finding it comfortable and roughly half finding it easy to put on (but more specific questions would be needed to determine what the children's evaluations were based on). Nonetheless, the absence of participant dropouts and the general tolerability and short application time of the headset support its feasibility, particularly if future studies address design aspects that may further improve comfort for younger populations. Moreover, as aperiodic activity has been linked to both typical and atypical cognitive development, these findings could inform the development of personalized interventions aimed at enhancing learning outcomes based on individual neural profiles. Future research should explore how classroom-based neuroimaging can be integrated into adaptive learning systems, early screening for math difficulties, and intervention programs tailored to individual cognitive profiles. In conclusion, our study highlights the potential of consumer-grade EEG as a viable tool for developmental cognitive neuroscience.

## Supplementary Information

Below is the link to the electronic supplementary material.

Supplementary file1 (DOCX 191 KB)

## Data Availability

The datasets generated during and/or analyzed during the current study are available in the Open Science Framework repository, https://osf.io/z52ux/?view_only=e76affb7cff14f0c93e3ca96ce60816d

## Code availability

The custom MATLAB, R, and Python scripts used for EEG preprocessing and aperiodic activity extraction are available from Open Science Framework (https://osf.io/z52ux/?view_only=e76affb7cff14f0c93e3ca96ce60816d).

## Declarations

### Open practices statement

This study was preregistered on the Open Science Framework (OSF): All data and analysis scripts are publicly available at https://osf.io/z52ux/.
