# Supplementary material for: Comparing aperiodic activity in consumer-grade and research-grade EEG: Reliability and association with mathematical ability
Source: Behav Res Methods. 2026 Jan 6;58(1):32. doi: 10.3758/s13428-025-02905-x (PMC12775036; doi:10.3758/s13428-025-02905-x)
Supplement: Supplementary file 1 — Supplementary file1 (DOCX 191 KB) [file 13428_2025_2905_MOESM1_ESM.docx]

**Supplementary Information**

**Table S1**

*Overview of EMOTIV and BioSemi recording setups*

| **Feature** | **EMOTIV EPOC X (consumer-grade)** | **BioSemi ActiveTwo (research-grade)** |
| --- | --- | --- |
| Electrode type | Water-based, fixed headset | Gel-based, active electrodes |
| Number of electrodes | 14 | 32 (subset F3/F4 used for comparison) |
| Sampling rate | 128 Hz (native) | 512 Hz (downsampled for analysis) |
| Referencing | Internal reference (EMOTIV default) | CMS/DRL reference |
| Environment | Schools (in a quiet room) | University lab, sound-attenuated |
| Setup time | ~15 minutes | ~30–45 minutes |
| Signal quality control | Visual indicators only (no impedance values) | Electrode impedances monitored (< ±25 mV) |
| Software used during recording | EMOTIVPro v4.6.5.580 | BioSemi ActiView v8.12 |
| Participants | N = 90, age 9–11 | N = 50, age 9–11 |
| Preprocessing | Same pipeline (FOOOF spectral decomposition) | Same pipeline (FOOOF spectral decomposition) |

*Note.* The BioSemi system does not measure electrode impedance in the conventional sense. The reported ‘offset’ values (kept below ±25 mV) reflect DC potential differences between each active electrode and the CMS reference, used to ensure stable recording conditions and avoid amplifier saturation.

**Table S2**

*General data quality metrics for EMOTIV and BioSemi recordings*

| **Metric** | **EMOTIV EPOC X** | **BioSemi ActiveTwo (children)** |
| --- | --- | --- |
| Electrode impedance / offset | Not available (visual only) | < ±25 mV (online monitored) |
| Mean power 49–51 Hz (µV²/Hz) | 0.00717 (range 0.000162–0.134) | 0.323 (range 0.0406–1.64) |
| Mean power 0.1–1 Hz (µV²/Hz) | 1.78e+03 (range 37.9–8.28e+04) | 454 (range 93.4–3.12e+03) |
| Proportion of segments removed (%) | 13.4% (range 0.0–35.3%) | 1.8% (range 0.0–18.4%) |
| Avg. number of usable segments | 151.3 (range 101–468) | 122.2 (range 111–171) |
| Proportion of participants excluded | 3 / 93 (3.2%) | 2 / 52 (3.8%) |

*Note.* Power values (e.g., 49–51 Hz, 0.1–2 Hz) are reported on a log10 scale; therefore, smaller numeric values correspond to higher absolute power.

**Supplementary Control Analysis with Matched Sample Sizes**

To ensure comparability across datasets, we repeated the reliability analyses for EMOTIV using a random subsample of 50 participants (to match the BioSemi sample size). The same procedure was used as in the main reliability analyses, computing intraclass correlations (ICCs) for offset and exponent parameters at F3 and F4 separately. The reliability of offset and exponent parameters in the EMOTIV subsample remained high and consistent with the results from the full sample (N = 90). Table S6 summarizes the ICCs for the EMOTIV full sample, the EMOTIV subsample (N = 50), and the BioSemi dataset (N = 50).

**Table S3**

*Intraclass correlations (ICCs) for aperiodic parameters in EMOTIV (full sample and subsample) and BioSemi*

| **Electrode–Parameter** | **EMOTIV Full (N = 90)** | **EMOTIV Subsample (N = 50)** | **BioSemi (N = 50)** |
| --- | --- | --- | --- |
| F3 Offset | 0.807 [0.721 – 0.869] | 0.817 [0.699 – 0.892] | 0.919 [0.861 – 0.953] |
| F3 Exponent | 0.938 [0.907 – 0.959] | 0.950 [0.915 – 0.972] | 0.919 [0.862 – 0.953] |
| F4 Offset | 0.760 [0.657 – 0.835] | 0.848 [0.748 – 0.911] | 0.902 [0.834 – 0.943] |
| F4 Exponent | 0.911 [0.862 – 0.943] | 0.896 [0.802 – 0.944] | 0.913 [0.852 – 0.949] |

To formally test differences between systems at matched sample sizes, we also performed a bootstrap comparison of ICCs between the EMOTIV subsample (N = 50) and BioSemi (N = 50). Results mirrored the full-sample analyses: exponent ICCs did not differ significantly between systems, and offset ICCs were lower for EMOTIV, but the 95% confidence intervals of the between-system difference included zero (Supplementary Table S7). This confirms that the observed reliability pattern is not driven by unequal sample sizes.

**Table S4**

*Bootstrap comparison of ICCs between EMOTIV subsample (N = 50) and BioSemi (N = 50)*

| **Electrode–Parameter** | **ICC EMOTIV** | **ICC BioSemi** | **ΔICC** | **95% CI** | **p-value** |
| --- | --- | --- | --- | --- | --- |
| F3 Offset | 0.817 | 0.919 | –0.101 | [–0.232, 0.025] | 0.130 |
| F3 Exponent | 0.950 | 0.919 | 0.031 | [–0.024, 0.097] | 0.266 |
| F4 Offset | 0.848 | 0.902 | –0.054 | [–0.216, 0.055] | 0.371 |
| F4 Exponent | 0.896 | 0.913 | –0.016 | [–0.115, 0.074] | 0.755 |

**Table S5**

*Bootstrap comparison of ICCs between EMOTIV and BioSemi (10,000 resamples)*

| **Electrode–Parameter** | **ICC_EMOTIV** | **ICC_BioSemi** | **ΔICC** | **95% CI** |
| --- | --- | --- | --- | --- |
| F3 Offset | 0.807 | 0.919 | –0.111 | [–0.224, 0.000] |
| F3 Exponent | 0.938 | 0.919 | +0.018 | [–0.029, 0.084] |
| F4 Offset | 0.760 | 0.902 | –0.142 | [–0.351, 0.022] |
| F4 Exponent | 0.911 | 0.913 | –0.002 | [–0.067, 0.078] |

**Table S6**

*Cross-validation errors and outlier-sensitivity for EMOTIV and BioSemi*

| **System** | **Electrode–Parameter** | **N** | **MAE** | **MedAE** | **P90AE** | **Drop_n** | **ΔMAE** | **95% CI** | **p-value** |
| --- | --- | --- | --- | --- | --- | --- | --- | --- | --- |
| EMOTIV | F3 Offset | 90 | 0.214 | 0.185 | 0.263 | 1 | 0.024 | [–0.025, 0.088] | 0.41 |
| EMOTIV | F3 Exponent | 90 | 0.198 | 0.177 | 0.297 | 3 | 0.028 | [–0.011, 0.072] | 0.18 |
| EMOTIV | F4 Offset | 90 | 0.198 | 0.171 | 0.260 | 1 | 0.022 | [–0.027, 0.084] | 0.45 |
| EMOTIV | F4 Exponent | 90 | 0.203 | 0.189 | 0.310 | 3 | 0.026 | [–0.012, 0.068] | 0.18 |
| BioSemi | F3 Offset | 50 | 0.095 | 0.095 | 0.116 | 3 | 0.010 | [–0.004, 0.025] | 0.16 |
| BioSemi | F3 Exponent | 50 | 0.103 | 0.099 | 0.128 | 3 | 0.013 | [–0.008, 0.034] | 0.23 |
| BioSemi | F4 Offset | 50 | 0.112 | 0.112 | 0.135 | 3 | 0.011 | [–0.004, 0.027] | 0.15 |
| BioSemi | F4 Exponent | 50 | 0.115 | 0.108 | 0.143 | 3 | 0.014 | [–0.006, 0.037] | 0.18 |

**Table S7**

*Session-stability of noise metrics (median [IQR]) in the first vs. second half of resting-state recordings*

| **System** | **Metric** | **Half 1** | **Half 2** | **Wilcoxon signed-rank p** |
| --- | --- | --- | --- | --- |
| EMOTIV | 49–51 Hz power | 0.00127 [0.00278] | 0.000529 [0.00143] | < .001 |
| EMOTIV | 0.1–1 Hz power | 313 [758] | 259 [472] | 0.003 |
| BioSemi | 49–51 Hz power | 0.203 [0.289] | 0.190 [0.268] | 0.037 |
| BioSemi | 0.1–1 Hz power | 308 [312] | 284 [277] | 0.703 |

**Supplementary Mediation Analysis**

To examine whether aperiodic activity measured with the EMOTIV EPOC X headset predicts mathematical ability and whether this relationship is mediated by working memory, we conducted a mediation analysis using Hayes’ PROCESS Model 4 with 10,000 bootstrap samples. Aperiodic activity (averaged offset and exponent) served as the independent variable, mathematical ability as the dependent variable, and verbal and visuospatial working memory as parallel mediators (see Figure S1). Prior to analysis, all continuous predictors were z-transformed to ensure comparability.

The mediation analysis revealed that the indirect effects of aperiodic activity on mathematical ability via verbal working memory (β = -0.01, 95% CI [-0.08, 0.04]) and visuospatial working memory (β = -0.02, 95% CI [-0.08, 0.004]) were not significant. Similarly, the total indirect effect (path c – c’) was not significant (β = -0.03, 95% CI [-0.12, 0.03]), indicating that working memory does not mediate the relationship between aperiodic activity and mathematical ability. However, both verbal working memory (path b1, β = 0.25, p = .013, 95% CI [0.05, 0.46]) and visuospatial working memory (path b2, β = 0.20, p = .051, 95% CI [-0.001, 0.40]) significantly and marginally significantly predicted mathematical ability, highlighting their relevance to math performance. The direct effect of aperiodic activity on mathematical ability (path c’) was marginally significant (β = -0.17, p = .072, 95% CI [-0.37, 0.01]), suggesting a weak negative association where low aperiodic activity relates to high mathematical ability. These findings suggest that while aperiodic activity is weakly associated with mathematical ability, its effect is not explained through working memory. Instead, verbal and visuospatial working memory independently contribute to mathematical performance, which aligns with our expectations.


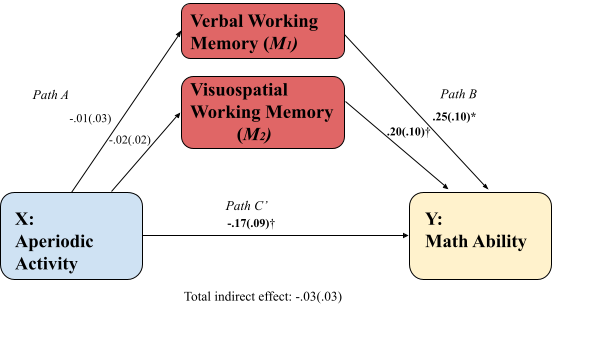


**Figure S1| Mediation model examining the relationship between aperiodic activity recorded with the EMOTIV EPOC X headset and mathematical ability, with verbal and visuospatial working memory as parallel mediators.** Path coefficients (β) are presented with standard errors in parentheses. The direct effect (path c') of aperiodic activity on math ability was marginally significant. Indirect effects through verbal and visuospatial working memory (paths a × b) were not significant, indicating no mediation. The total indirect effect was also non-significant. Significance levels: p < .05, †p < .10.

**Supplementary Correlation Comparison (Fisher’s Z)**

To formally test whether the relation between aperiodic activity and mathematical ability differed across datasets, we compared the independent correlations using Fisher’s Z transformation. The correlation for the EMOTIV dataset was *r* = –.225 (95% CI [–.414, –.018], N = 89), and for the BioSemi dataset *r* = –.027 (95% CI [–.306, .256], N = 49). Fisher’s Z-test indicated that the correlation strengths did not differ significantly between the two datasets (*Z* = –1.106, *p* = .269).

**Supplementary Comfort Questionnaire Results**

**Table S8**

*Question 1 Comfort Questionnaire: Did you find the headset comfortable?*

|  | **Frequency** | **Percentage** |
| --- | --- | --- |
| **Yes** | 61 | 65.60 |
| **No** | 32 | 34.40 |
| **Total** | 93 | 100.00 |

**Table S9**

*Question 2 Comfort Questionnaire: Was the headset easy to put on?*

|  | **Frequency** | **Percentage** |
| --- | --- | --- |
| **Yes** | 44 | 48.90 |
| **No** | 46 | 51.10 |
| **Total** | 93 | 100.00 |

**Table S10**

*Question 3 Comfort Questionnaire: Did the headset fit too tight, too loose, or just right?*

|  | **Frequency** | **Percentage** |
| --- | --- | --- |
| **Just right** | 73 | 78.90 |
| **Too loose** | 4 | 4.40 |
| **Too tight** | 16 | 16.70 |
| **Total** | 93 | 100.00 |

**Supplementary Comparison of Spectral Fits**

Independent t-tests revealed no significant differences in R² values between EMOTIV and BioSemi for either F3 (*t*(4) = 0.06, *p* = .955) or F4 (*t*(4) = 0.45, *p* = .676), suggesting comparable model fits across systems. Similarly, MAE was not significantly different between EMOTIV and BioSemi for both F3 (*t*(4) = 0.71, *p* = .515) and F4 (*t*(4) = 0.74, *p* = .502), indicating that systematic differences in power spectrum estimates were not observed. These findings suggest that the two EEG systems yield comparable spectral model fits and estimation accuracy.


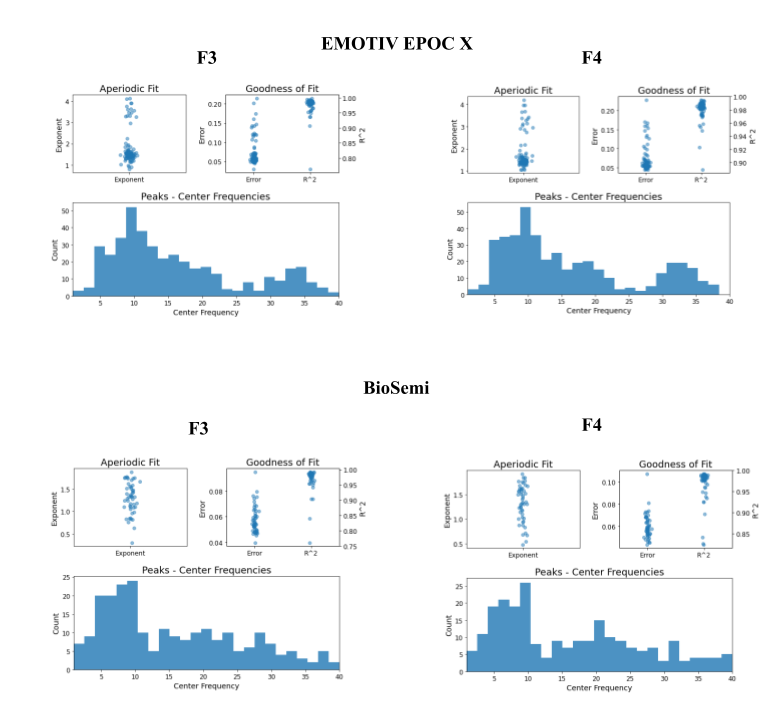


**Figure S2| Comparison of aperiodic fit, model goodness-of-fit metrics, and peak frequency distributions between the EMOTIV EPOC X (top row, N = 90) and BioSemi (bottom row, N = 50) EEG systems for electrodes F3 (left) and F4 (right).** The aperiodic fit scatter plots show the estimated exponent values across participants. The goodness-of-fit plots display the distribution of model error (MAE) and R² values, indicating high model fit quality for both systems. The histograms represent the distribution of peak center frequencies.


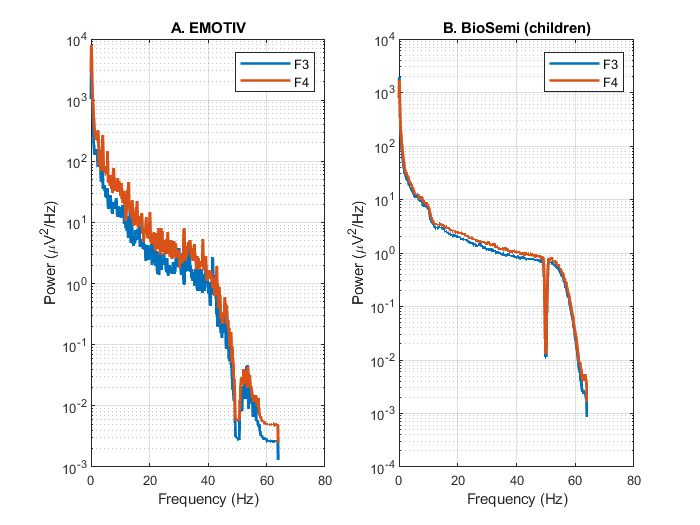


**Figure S3. Overlay of mean power spectral densities (PSDs) of the children for EMOTIV and BioSemi EEG systems.** Panel A shows EMOTIV recordings (F3, F4), panel B shows BioSemi recordings (F3, F4). PSDs are averaged across participants (N = 90 in EMOTIV sample and N = 50 in BioSemi sample) and plotted on a log–log scale. Both systems capture the expected 1/f structure of neural activity. Compared to BioSemi, EMOTIV recordings exhibited greater variability in the higher-frequency range and less distinct periodic peaks, while the BioSemi data showed smoother spectra and a clear 50 Hz notch. These differences illustrate general data quality characteristics complementing the quantitative metrics in Table S2.
